# Assistive Technology to Support Dementia Management: Protocol for a Scoping Review of Reviews

**DOI:** 10.2196/57036

**Published:** 2024-11-11

**Authors:** Chaitali Desai, Erica Dove, Jarshini Nanthakumar, Emilia Main, Heather Colquhoun, Arlene Astell, Alex Mihailidis, Natasha Layton, Amer M Burhan, Brian Chan, Rosalie H Wang

**Affiliations:** 1 Rehabilitation Sciences Institute Temerty Faculty of Medicine University of Toronto Toronto, ON Canada; 2 KITE Research Institute Toronto Rehabilitation Institute University Health Network Toronto, ON Canada; 3 Department of Occupational Science & Occupational Therapy Temerty Faculty of Medicine University of Toronto Toronto, ON Canada; 4 Library and Information Services University Health Network Toronto, ON Canada; 5 Department of Psychology Northumbria University Newcastle upon Tyne United Kingdom; 6 Rehabilitation, Ageing and Independent Living (RAIL) Research Centre Faculty of Medicine, Nursing and Health Sciences Monash University Melbourne Australia; 7 Australian Rehabilitation and Assistive Technology Association (ARATA) Beaumaris Australia; 8 Department of Psychiatry Temerty Faculty of Medicine University of Toronto Toronto, ON Canada; 9 Ontario Shores Centre for Mental Health Sciences Whitby, ON Canada; 10 Toronto Dementia Research Alliance Toronto, ON Canada; 11 Institute of Health Policy, Management and Evaluation University of Toronto Toronto, ON Canada

**Keywords:** assistive technology, assistive products, dementia, care partners, caregivers, elderly

## Abstract

**Background:**

In Canada, more than 60% of persons living with dementia reside in their own homes, and over 25% rely heavily on their care partners (ie, family members or friends) for assistance with daily activities such as personal hygiene, eating, and walking. Assistive technology (AT) is a key dementia management strategy, helping to maintain health and social support in home and community settings. AT comprises assistive products and services required for safe and effective use. Persons living with dementia and their care partners often require multiple types of AT to maintain their needs, dignity, and autonomy. AT for dementia management is rapidly developing with abundant scientific literature, which can present a challenge to efficiently navigate and extract insights for policy and personal decision-making.

**Objective:**

This scoping review aims to synthesize review-level evidence from published scientific literature on AT to support dementia management for persons living with dementia and their care partners in their homes and communities. Research gaps in knowledge and areas for further investigation into the use and access of AT will be identified. This review will provide an overview of AT types and characteristics and chart the outcomes and conclusions in review-level evidence.

**Methods:**

This review will follow the Joanna Briggs Institute’s framework for conducting scoping reviews and the PRISMA-ScR (Preferred Reporting Items for Systematic Reviews and Meta-Analyses extension for Scoping Reviews) guidelines. In total, 6 electronic databases will be searched. Articles will be screened according to the “Population-Concept-Context (PCC)” framework for eligible studies. Population includes persons living with dementia, their care partners, and health care professionals (eg, therapists or others who recommend AT). Concept includes AT and self-help devices of many types. Context includes homes and communities. A data charting template will guide data extraction, charting, and summarization. A descriptive numerical summary and an overview of the findings will be presented. Data, such as (1) article information (eg, author and year), (2) article characteristics (eg, review type), (3) AT types and characteristics, (4) setting and population characteristics, and (5) key review outcomes and conclusions, will be extracted.

**Results:**

A total of 10,978 unique citations were identified across the 6 electronic databases. This review is in the full-text screening stage, which is expected to be completed by October 2024.

**Conclusions:**

This review will provide a comprehensive understanding and documentation of the published scientific literature on AT to support dementia management. Findings from this review are expected to provide evidence-based insights on the complexities of AT types, uses, availability, and access. The author group’s diverse national and international perspectives may contribute to knowledge exchange and influence standards to improve the daily function, safety, and well-being of persons living with dementia.

**Trial Registration:**

Open Science Framework DKSM9; https://osf.io/dksm9

**International Registered Report Identifier (IRRID):**

PRR1-10.2196/57036

## Introduction

### Background

As the population ages, the number of older adults affected by dementia, most commonly being Alzheimer disease, is expected to triple by 2050 [1]. Age is a significant risk factor for dementia [1]. According to the *Diagnostic and Statistical Manual of Mental Disorders, Fifth Edition, Text Revision* (*DSM-5-TR*), dementia, a major neurocognitive disorder, refers to a significant decline over time in one or more cognitive domains (“executive function, complex attention, memory, language, learning perceptual-motor, social cognition”) [2]. The degree of dementia severity is determined by its impact on the ability of persons living with dementia to independently carry out activities of daily living [2,3]. In Canada, more than 60% of persons living with dementia reside in their own homes, and over 25% rely heavily on their care partners (ie, family members or friends) for assistance with daily activities such as personal hygiene, eating, and walking [4]. However, care partners face challenges in supporting persons living with dementia due to competing demands (eg, children and jobs), resulting in around two-thirds of care partners experiencing stress and depressive symptoms [5]. To effectively manage dementia at home and cope with symptoms, persons living with dementia and their care partners need diverse strategies and interventions beyond medication alone [1,6-8]. With an aging population, the risks of developing dementia pose a major public health concern. As such, there is heightened interest and urgency for strategies and interventions to maintain health and social support for persons living with dementia and their care partners. Assistive technology (AT) is a key dementia management strategy [9], enabling persons living with dementia to live at home longer [4,10] and contributing to maintained health and social services in home and community settings [11].

### Assistive Technology

AT is the umbrella term for the combination of assistive products and the services needed to ensure safe assessment, distribution, and use of assistive products [12,13]. As defined by the International Organization for Standardization (ISO), assistive products (ie, devices, software, equipment, or instruments) maintain or improve a person’s functionality and reduce disability [14]. The services associated with AT refer to the “human factors” necessary to fit the product to the person, environment, and task, and include the application of organized knowledge and skills to the provision, use, and assessment of assistive products [12,15,16]. AT is most effective when provided with “early intervention, careful assessment, the correct prescription, and home-based follow-up training in how to use AT” [17]. AT is closely associated with environmental interventions (eg, home modifications), forming a technology chain of supports [18]. There has been rapid development of technological supports for older people and people with disabilities, including for those with functional limitations due to dementia [19]. Persons living with dementia and their care partners often require multiple types of AT to meet their needs and maintain their dignity and autonomy. AT includes products such as a wheelchair, medication reminder, appliance monitor, wayfinding device, and mobile app for navigation assistance [17], involving both access to the product and assessment, trial, training, maintenance, and follow-up services [15].

### Rationale

A growing body of research is focused on developing, evaluating, and understanding AT applications for dementia management to promote the well-being of persons living with dementia and alleviate the strain experienced by their care partners [10,11,20-22]. For example, monitoring technologies for persons living with dementia have been found to increase feelings of safety and security, promote their independence, and reduce care partner strain [10,23-27]. Reminder systems have been shown to increase the execution of daily activities such as taking medications, eating and drinking [10,28-31]. AT that target safety in the homes of persons living with dementia have been observed to reduce falls and accidents and decrease care partner strain [32]. Information and communication technologies (eg, mobile devices, apps, and the internet) have been used to support cognitive stimulation and emotional well-being in persons living with dementia and social support and education for care partners [10]. The abundance of diverse and broad-ranging published reviews, compounded by the rapid development of technological supports for older people and people with disabilities [19], presents a challenge for researchers, policy makers, persons living with dementia and their care partners, and health care professionals (eg, therapists) who recommend AT for dementia management, to efficiently navigate and extract valuable insights for policy and personal decision-making regarding AT to support dementia management. Therefore, a scoping review of reviews is warranted to comprehensively, systematically, and feasibly map the scientific literature in this field.

### Review Objectives

This paper outlines a protocol for a scoping review of reviews that aims to synthesize review-level evidence from published scientific literature on AT to support dementia management for persons living with dementia and their care partners in their own homes and communities. This synthesis will identify research gaps in knowledge and areas for further investigation regarding the use and access of AT by persons living with dementia, their care partners, and health care professionals (eg, therapists) who recommend AT for dementia management. This review will also chart the key outcomes used and conclusions made in review-level evidence.

## Methods

### Terminology, Definitions, and Classifications

#### Dementia

While age is a significant risk factor for dementia [1], cognitive decline can also impact younger individuals [2]. The recent versions of the *Diagnostic and Statistical Manual of Mental Disorders* (*Diagnostic and Statistical Manual of Mental Disorders, Fifth Edition* [*DSM-5*] and *DSM-5-TR*) criteria have updated the definition to “major neurocognitive disorder,” offering a broader perspective [2] of the conditions that fall under the umbrella of major neurocognitive disorder (previously known as dementia). The evolving understanding of dementia in *DSM-5-TR* will guide the screening process, providing this review with more relevant and applicable articles. However, considering the term “dementia” continues to be used in society and medical literature [2,33], it will be used in this review.

#### Assistive Technology

AT for persons living with dementia has rapidly developed, and the scope of this field is broad, diverse, and extensive in Canada and beyond. Therefore, subjective interpretations may arise when defining, categorizing, or classifying AT for persons living with dementia. There is inconsistency in the terminology used in the literature and geographical regions in relation to AT [34]. The literature often uses vague and interchangeable terms such as AT, products, services, devices, technologies, equipment, aids, and supports. However, the term “assistive products” is now preferred and commonly used internationally, including the World Health Organization’s (WHO) [11] and ISO’s [14] definitions. In this protocol, we will substitute the term “AT” used in previous literature with “assistive products” when it is clear that only products, not services, are being referenced.

#### Assistive Products—Classification and Terminology (ISO 9999:2022)

There are several classifications for AT interventions for persons with disabilities, such as the global standardized nomenclature for medical devices (Global Medical Device Nomenclature [GMDN] and Systematized Nomenclature of Medicine [SNOMED]) [35], WHO’s International Classification of Functioning, Disability and Health (ICF) [36], and ISO 9999:2022 Assistive Products—Classification and Terminology [14]. Although standards are regularly updated, as in WHO ICF [37], and the definition of disability encompasses (ie, does not exclude) age and progressive conditions such as dementia, there is no focal sublist of classifications focused exclusively on dementia [38]. ISO 9999 is a related member of the WHO Family of International Classifications (FIC) [14], which provides high-quality classifications for relevant health sectors [37]. Therefore, it is most appropriate to use the ISO 9999 taxonomy in categorizing or classifying the types and characteristics of AT encountered in this review, providing this review with precise guidance [39].

ISO 9999 defines 11 classes, subclasses, and divisions of assistive products that are most relevant to optimizing functioning and reducing disability [14] (refer to Table 1 for the 11 classes of assistive products). This approach proves especially valuable when many countries use varying names to refer to the same product category and help specify definitions and identify the conceptual boundaries [40,41] of the AT types and characteristics important to manage dementia at home. This approach also aligns with the work carried out in Australia by one of the coauthors of this review (NL), offering an international perspective.

**Table 1:** International Organization for Standardization (ISO) 9999:2022 classes of assistive products.

Code	Title
04	“Assistive products for measuring, stimulating or training physiological and psychological functions”
06	“Orthoses and prostheses”
09	“Assistive products for self-care activities and participation in self-care”
12	“Assistive products for activities and participation relating to personal mobility and transportation”
15	“Assistive products for domestic activities and participation in domestic life”
18	“Furnishings, fixtures and other assistive products for supporting activities in indoor and outdoor human-made environments”
22	“Assistive products for communication and information management”
24	“Assistive products for controlling, carrying, moving and handling objects and devices”
27	“Assistive products for controlling, adapting or measuring elements of physical environments”
28	“Assistive products for work activities and participation in employment”
30	“Assistive products for recreation and leisure”

### Scoping Review of Reviews

The protocol design was initially informed by establishing the need for a scoping review of reviews (instead of a scoping review). The information specialists (Maureen Pakosh and EM) from the Department of Library and Information Services at the University Health Network, with the first author (CD), conducted an exploratory literature search on AT to support dementia management. Following this search, CD consulted with the principal investigators (RHW and BC) to clarify and establish the review question, Population-Concept-Context (PCC) framework for eligible studies [42,43], and design of this review. Due to the abundance of diverse and broad-ranging published reviews found in this topic area, a scoping review of reviews was selected to map the scientific literature comprehensively, systematically, and feasibly on AT to support dementia management.

This review will follow the Joanna Briggs Institute (JBI) methodological framework and guidance for conducting scoping reviews [42] and the PRISMA-ScR (Preferred Reporting Items for Systematic Reviews and Meta-Analyses Extension for Scoping Reviews) checklist for reporting results [42,43]. This review will use the PRISMA-ScR sections that are consistent with our review of reviews and justification for areas that are not reported will be presented in the final review article. The main elements of this scoping review framework include (1) review question, (2) search strategy, (3) inclusion and exclusion criteria, (4) sources of evidence selection, (5) data charting, and (6) analysis of the evidence and presentation of results. A consultation process with diverse interest-holder groups to validate and disseminate findings will be included in this review. Guidance for the exploratory review question and broad search for relevant studies will come from the PCC framework for eligible studies [42,43]. As the main objective of this review is to describe the state of the scientific literature, a quality assessment will not be conducted [40,42,44-46].

This scoping review protocol is registered with the Open Science Framework (OSF) [47]. When conducting this review, any protocol amendments will be documented in the OSF and final paper. Once this review is complete, the results will be disseminated through publications and conferences on AT, health care policies, and dementia topics.

### Review Question

This scoping review of reviews will be guided by the question, “What AT types and characteristics have been documented in the scientific literature to support dementia management for persons living with dementia and their care partners in their own homes and communities?”

The review question is formulated using the PCC framework for eligible studies [42,43] (Table 2).

**Table 2:** The Population-Concept-Context (PCC) framework for eligible studies.

Concept	Determinants
Population (P)	Persons living with dementia Care partners of persons living with dementia Health care professionals (eg, therapists) who recommend AT^a^ to persons living with dementia and their care partners for dementia management
Concept (C)	AT types and characteristics to support dementia management
Context (C)	Persons living with dementia and their care partners in their own homes and communities

^a^AT: assistive technology.

### Search Strategy

The search strategy will include a previous review of relevant literature by the first author (CD), informing the initial list of search terms and strategy. Subject matter experts (AA, AM, BC, HC, and RHW) and 2 information specialists (Maureen Pakosh and EM) will provide additional inputs and revisions. The search strategy, subject headings, and corresponding free-text terms will be informed by the priority assistive products list [13], the ISO 9999 classification and terminology standard for assistive products [14], and My AT Outcomes Framework (MyATOF) [8]. The priority assistive products list was introduced by the WHO as the first stage of implementing a global commitment to improve access to assistive products—the Global Cooperation on Assistive Technology (GATE) [13]. MyATOF is an Australian framework that integrates AT process principles and outcomes research, directing interest-holders in articulating AT usage through 6 dimensions (ie, supports, outcomes, costs, rights, service delivery, and consumer experience) [48].

The search scope will remain broad by using 2 PCC concepts (population and concept) from the PCC framework in Table 2. As this scoping review will not compare AT interventions with other dementia management strategies (eg, medical devices and treatments for dementia) [49], no comparison terms will be incorporated into the search.

The initial iterations of the search included synonyms of AT (eg, self-help devices) and terms related to technology (eg, computers and electronics). However, since AT is a broad concept, additional terms will be generated through review of the above sources and discussion among the research team to ensure comprehensive representation of AT products and functional abilities addressed by AT. In addition to types of products (eg, computers and wheelchairs), terms combining functional activities (eg, eating and drinking) and technology (eg, device, product, and tool) will provide additional sensitivity to the search (Multimedia Appendix 1). Using this strategy limits the need to include all potential products (eg, potato peelers), which would be impractical and unlikely to yield results in the scientific literature.

The search process will be conducted in collaboration with Maureen Pakosh and EM. Maureen Pakosh will develop the initial draft search strategies in 6 electronic databases, including APA PsycINFO (Ovid), CINAHL Ultimate (EBSCOhost), Embase (Ovid), Emcare Nursing (Ovid), Medline ALL (Ovid), and Web of Science Core Collection. Decisions regarding the search terms and the search process will be resolved over multiple iterations of the search strategy through discussion among the research team (AA, AM, AMB, BC, CD, ED, EM, HC, JN, NL, and RHW).

Following additional consultation with diverse interest-holder groups (refer to *Consultation Process* under the *Methods* section), EM will revise the initial MEDLINE search strategy, as necessary. The MEDLINE search strategy will be peer-reviewed by a third rehabilitation information specialist (Jessica Babineau) following PRESS (Peer Review of Electronic Search Strategies) guidelines [50]. A study design filter will be included to limit the results to reviews. No date or language restrictions will be imposed on the search; however, non-English results will be excluded during the title, abstract, and full-text screening stages.

Based on the MEDLINE search strategy, EM will conduct corresponding searches in the 6 electronic databases above. The search will include articles from database inception to July 2023. Bibliographic duplicates will be removed by EM using EndNote 20 (Clarivate) [51] and an adapted Bramer method [52,53]. Deduplicated results will be uploaded into Covidence (Veritas Health Innovation Ltd), a software platform designed for screening and data extraction [54], by CD.

### Inclusion and Exclusion Criteria

The screening process will be guided by the PCC inclusion and exclusion criteria (Textbox 1).

**Inclusion and exclusion criteria.**

**Inclusion criteria**

- Population
  Persons living with dementia (dementia, as a major neurocognitive disorder, is defined as cognitive decline over time in one or more cognitive domains [“executive function, complex attention, memory, language, learning, perceptual-motor, social cognition”] [2] and interference with independence in daily activities such as managing finances or medication [2]. Dementia may be due to a range of subtypes such as “Alzheimer’s disease, frontotemporal degeneration, Lewy body disease, vascular disease, traumatic brain injury, substance or medication use, HIV infection, prion disease, Parkinson’s disease, Huntington’s disease” [2].)
  Care partners (ie, family members or friends) of persons living with dementia
  Health care professionals (eg, therapists) who recommend assistive technology (AT) to persons living with dementia and their care partners for dementia management
  Persons living with dementia and care partners of any age, gender, or ethnicity, living in any country or region
- Concept
  AT, that is, assistive products (devices, software, equipment, or instruments) and associated services that maintain or improve a person’s functioning and independence [14]
  AT that falls under the 11 classes of assistive products classified by International Organization for Standardization (ISO) 9999 [14] (Table 1)
- Context
  Persons living with dementia living in their own home and community (a home and community is where an individual can live safely, independently, and comfortably [55] as they age, with access to health and social support [56].)

**Exclusion criteria**

- Population
  Persons living without dementia
  Persons living with cognitive decline not due to dementia
  Persons living with cognitive decline exclusively due to mild cognitive impairment or mild neurocognitive disorder (mild cognitive impairment, or mild neurocognitive disorder, is defined as a modest impairment in several cognitive domains and not interfering with independence in daily activities [2].)
  Persons living with cognitive decline exclusively due to delirium (delirium is defined as “disturbance in attention (ie, reduced ability to direct, focus, sustain, and shift attention)” [2], with reduced environmental awareness, over a short period of time [2].)
  Persons living with cognitive decline exclusively due to another mental disorder such as major depressive disorder, schizophrenia, etc [2]
- Concept
  AT used for prevention, assessments, monitoring, diagnosis, and treatment will not be included unless the AT is used to help persons living with dementia maintain or improve functioning and independence in their daily lives
- Context
  Persons living with dementia living exclusively in a shared communal environment (shared communal environments offer 24-hour, 7-day-a-week supervised on-site care, including health care, personal care, meals, laundry, and housekeeping [57]. Among these are long-term care homes and facilities, known within Canada as “nursing homes, personal care facilities, residential continuing care facilities, etc.” [55])
  Hospitals, primary care ambulatory or outpatient clinics and doctor’s offices

### Types of Evidence Sources

Articles of any type of review methodology (eg, scoping review, systematic review, and narrative review) published in peer-reviewed journals will be included. Therefore, researchers with expertise in both quantitative and qualitative methodologies will contribute to the development of the narrative. Non–peer-reviewed articles, protocols, theses or dissertations, editorials, conference abstracts, gray literature, and non-English articles will be excluded.

### Source of Evidence Selection

#### Title and Abstract Screening

A total of 3 reviewers (CD, ED, and JN) will be involved in the title and abstract screening process. The first author (CD) will independently screen all eligible articles in Covidence [46] to determine their suitability for a full-text review according to the PCC inclusion and exclusion criteria. Subsequently, each secondary reviewer (ED and JN) will screen a subset of articles independently. CD will monitor the level of agreement between the reviewers throughout the screening process. Covidence will export the agreement proportion to Microsoft Excel. Designating each article into yes, no, or maybe categories and the reasons for these designations will be recorded in Covidence [54]. Predefined tags in Covidence will be created to ensure ease of recording reasons for exclusion throughout the screening process. The reviewers will meet throughout the process to discuss their decisions and reasons for including or excluding articles. Any discrepancies will be resolved in discussion with the principal investigators (BC and RHW). The JBI recommended procedure for pilot testing will be used for the title and abstract screening stage [42]. CD, in collaboration with the research team, will critically analyze the final sample of articles sent to full-text screening to inform the creation of the data charting template. The data charting template will be developed in Microsoft Excel [58].

#### Full-Text Screening

In total, 3 reviewers (CD, ED, and JN) will be involved in the full-text screening process. The first author (CD) will independently conduct the screening in Covidence [54] to determine their inclusion in the scoping review. Subsequently, each secondary reviewer (ED and JN) will screen a subset of full-text articles independently. CD will critically analyze the final sample of studies, and all reviewers will be involved in the charting process. At the full-text screening stage, the reviewers may request further details about an article directly from the corresponding author. The number of articles reviewed and included will be outlined in a PRISMA-ScR flow diagram [59], with reasons for exclusion at the full-text level. The reviewers will continue to meet throughout to discuss their decisions and reasons for including or excluding articles. Discussions with the principal investigators (BC and RHW) will occur to resolve any discrepancies. The JBI-recommended procedure for pilot testing will be used for the full-text screening stage [42]. CD, in collaboration with the research team, will critically analyze the final sample of articles sent for data charting to inform the revision of the data charting template if necessary.

### Data Charting

Once the articles are fully reviewed, the data will be extracted, charted, and integrated into a comprehensive format (ie, table). The data charting template will be used to capture relevant data from included articles to meet the objectives of this scoping review. An example of the data to be extracted is described in Table 3.

**Table 3:** Data extraction template.

Type of data	Description of data
Article information	Author, publication year, country, journal name, and location of publication.
Article characteristics	Review type, number of studies included in the review, and reported time period of studies included.
AT^a^ types and characteristics	All AT types and characteristics described in the review, and the purpose of AT described for and used by persons living with dementia, their care partners, and health care professionals (eg, therapists) who recommend AT to persons living with dementia and their care partners.
Setting and population characteristics	Country or region, type of home and community setting described where AT is used, available or recommended. Age, sex, gender, or ethnicity. Given women’s higher risk for dementia [60] and increased likelihood to be care partners [61], participants’ sex and gender, and gender-related factors influencing AT use and access, will be collected [60,61].
Key review outcomes and conclusions	Main findings, outcomes, and conclusions drawn from each article.

^a^AT: assistive technology.

The JBI-recommended procedure for pilot testing will be used for data extraction [42]. A total of 3 reviewers (CD, ED, and JN) will independently conduct a pilot test to extract data from at least 3 full-text articles. The pilot test findings will be refined and adjusted through team discussions. The pilot test will continue until reviewers adequately understand the data charting template, context, and process of data extraction.

Following the completion of the pilot test, reviewers will proceed to extract data from all full-text articles using the data charting template. If specific information is unavailable, reviewers may contact the study authors to obtain it. As the review progresses, the reviewers may extract additional information of relevance to address the scoping review objectives and revise the data charting template accordingly [42]. In conducting this scoping review, the focus is on synthesizing and analyzing existing reviews. Therefore, the scope of this review will not include an in-depth analysis of individual primary studies.

### Analysis of the Evidence and Presentation of Results

After extracting the data from included full-text articles, the data will be synthesized using descriptive analysis (eg, frequencies, means, and ranges). A descriptive numerical summary and overview of the findings will be presented in tabular format.

### Consultation Process

The Project Advisory Committee (PAC), comprising persons living with dementia, care partners, researchers, partner and network organization representatives, and knowledge user partners who have a direct interest in the outcomes of this research [62], will be consulted for their inputs. They will also provide resources to complete this scoping review and assist in knowledge dissemination strategies and activities. The PAC convenes every 4 to 6 months. PAC members will also be consulted individually on matters where there is expertise in content, methodology, analyses, and knowledge mobilization and dissemination.

### Ethical Considerations

This review does not involve collecting any data from human or animal participants; therefore, research ethics approval is unnecessary.

## Results

Across the 6 electronic databases (APA PsycINFO [Ovid], CINAHL Ultimate [EBSCOhost], Embase [Ovid], Emcare Nursing [Ovid], Medline ALL [Ovid; includes PubMed non-MEDLINE records], and Web of Science Core Collection), 21,747 results were retrieved. After deduplication, 10,978 unique citations were retained. A search strategy for MEDLINE is provided in Multimedia Appendix 1. This review is in the full-text screening stage, which is expected to be completed by October 2024.

## Discussion

### Anticipated Findings

This review will provide a comprehensive understanding and documentation of the published scientific literature on AT to support dementia management. Findings from this review are expected to provide evidence-based insights to diverse groups of interest-holders to facilitate discussions on the complexities of current AT types, uses, availability, and access for persons living with dementia and their care partners. Furthermore, the author group’s diverse national and international perspectives may contribute to knowledge exchange and influence standards to improve the daily function, safety, and well-being of persons living with dementia.

Findings from this review will also lay the groundwork for a larger Canadian Institute of Health Research (CIHR)–funded project to examine the creation and economic evaluation of combining different AT into effective bundles or packages [15,63-67] for dementia management in Canada and beyond. This work aligns with national initiatives such as the Canadian Dementia Strategy [68] and global recommendations by the WHO regarding the formulation of a comprehensive “Package of Interventions for Rehabilitation” for health conditions, including dementia [69].

The field of AT for dementia management continues to grow [10,11,19-22], with an abundance of diverse and broad-ranging published reviews focused on developing, evaluating, and understanding AT applications. Despite its considerable impact on the field, there are still challenges to efficiently navigate and extract valuable insights regarding AT for policy and personal decision-making. This scoping review of reviews is therefore warranted to map the scientific literature in this area comprehensively, systematically, and feasibly.

### Limitations

This scoping review may have limitations. First, this review does not incorporate searches of gray literature and other non–peer-reviewed academic resources. As the development of AT is rapidly progressing, these advancements may not be documented in the existing scientific literature. In addition, only review articles will be considered for inclusion, and if there are AT interventions reported exclusively in primary reports, they may not be captured in this review. An independent environmental scan will be conducted by members of the research team (CD, BC, JN, and RHW) as a separate review, encompassing various documents and resources not typically accessible through scientific databases. These documents and resources may include organizational websites, policy documents, reports, magazine articles, newsletters, and blogs. Second, the search strategy may miss relevant reviews due to the selected databases lacking coverage of all published literature, inaccuracies in database indexing, and the broad scope of AT and complexity and limitations of terms used [70]. This limitation is addressed by searching multiple bibliographic databases and using a comprehensive set of search terms covering both products and relevant functional abilities. In addition, to mitigate these risks, a hand-search of the reference lists of relevant articles will be considered to identify any further citations that may have been overlooked during the initial electronic database search. However, the reviews may exclude other languages as well. It is also possible that the search strategy may limit the types of AT that appear in the results because the search terms are based on established terminology and categories of AT. Finally, only studies written in English will be included, which may exclude relevant reviews from non–English-speaking countries or regions, limiting our findings’ generalizability and applicability.

Although limitations may exist, this scoping review will enable a comprehensive understanding and documentation of the types and characteristics of AT to support dementia management for persons living with dementia and their care partners in their own homes and communities.

### Conclusions

The knowledge synthesized from this review may broadly serve as a framework to create greater insights into dementia management using AT [61] among persons living with dementia; their care partners; and various other interest-holders groups, such as health care professionals (eg, therapists) who recommend AT to persons living with dementia and their care partners, policy makers, researchers, partner organizations, and networks. It is also anticipated that the research community may benefit from the generated knowledge from this review to formulate new research inquiries and approaches for larger-scale studies on AT use and access by persons living with dementia and their care partners for dementia management.

## Appendix

Multimedia Appendix 1 MEDLINE search strategy.

## Abbreviations

AT: assistive technology
CIHR: Canadian Institute of Health Research
DSM-5: Diagnostic and Statistical Manual of Mental Disorders, Fifth Edition
DSM-5-TR: Diagnostic and Statistical Manual of Mental Disorders, Fifth Edition, Text Revision
FIC: Family of International Classifications
GATE: Global Cooperation on Assistive Technology
GMDN: Global Medical Device Nomenclature
ICF: International Classification of Functioning, Disability and Health
ISO: International Organization for Standardization
JBI: Joanna Briggs Institute
MyATOF: My AT Outcomes Framework
OSF: Open Science Framework
PAC: Project Advisory Committee
PCC: Population-Concept-Context
PRESS: Peer Review of Electronic Search Strategies
PRISMA-ScR: Preferred Reporting Items for Systematic Reviews and Meta-Analyses Extension for Scoping Reviews
SNOMED: Systematized Nomenclature of Medicine
WHO: World Health Organization
