# Supplementary material for: Assistive Technology to Support Dementia Management: Protocol for a Scoping Review of Reviews
Source: JMIR Res Protoc. 2024 Nov 11;13:e57036. doi: 10.2196/57036 (PMC11589495; doi:10.2196/57036)
Supplement: Multimedia Appendix 1 [file resprot_v13i1e57036_app1.docx]

Database: Ovid MEDLINE(R) ALL <1946 to July 11, 2023>

| # | Query | Results from 12 Jul 2023 |
| --- | --- | --- |
| 1 | Neurocognitive Disorders/ or exp Cognition Disorders/ or exp Dementia/ | 290,237 |
| 2 | exp Parkinsonian Disorders/ or Lewy Bodies/ | 98,284 |
| 3 | (dementia* or predementia* or parkinson* or lewy bod* or alzheimer* or binswanger* or pick* disease or supranuclear palsy or senil*).ti,ab,kf,jw. | 413,573 |
| 4 | ((frontotemporal or fronto temporal or small vessel) adj3 (disease* or disorder* or degenerat*)).ti,ab,kf,jw. | 12,252 |
| 5 | ((cogniti* or neurocogniti* or neurodegenerat* or memory) adj3 (disease* or impairment* or dysfunction* or disorder* or declin* or deteriorat* or degenerat*)).ti,ab,kf,jw. | 278,379 |
| 6 | or/1-5 [Dementia and Related Disorders] | 656,489 |
| 7 | exp Self-Help Devices/ | 13,242 |
| 8 | exp Technology/ or exp Computer Systems/ or exp Software/ or Video Games/ or exp Artificial Intelligence/ or Robotics/ | 946,535 |
| 9 | telemedicine/ or telerehabilitation/ | 38,354 |
| 10 | exp Wearable Electronic Devices/ or exp Sensory Aids/ or Brain-Computer Interfaces/ | 36,241 |
| 11 | Reminder Systems/ or Telephone/ or exp Cell Phone/ or Diaries as Topic/ | 38,332 |
| 12 | ((assist* or adapt* or rehab* or disab* or self manag* or self help or compensat*) adj3 (technolog* or device* or product* or tool* or instrument* or equipment or gadget* or app or apps or system or systems or solution* or software or service* or bundle or package* or aid* or assistan* or support* or self manag*)).ti,ab,kf,jw. | 267,831 |
| 13 | ((memory or cueing or reminder* or remember* or prompt* or sensory or vision or visual or hear or hearing or communicat*) adj3 (technolog* or device* or product* or tool* or instrument* or equipment or gadget* or app or apps or system or systems or solution* or software or service* or bundle or package* or aid* or assistan* or support* or self manag*)).ti,ab,kf. | 121,924 |
| 14 | ((smart* or electronic or digital or virtual or robot* or voice or automat* or motion or wearable) adj3 (technolog* or device* or product* or tool* or instrument* or equipment or gadget* or app or apps or system or systems or solution* or software or service* or bundle or package* or aid* or assistan* or support* or self manag*)).ti,ab,kf. | 185,614 |
| 15 | ((mobil* or portable or surveillance or track* or locat* or monitor* or safety or alert* or alarm* or signal*) adj3 (technolog* or device* or product* or tool* or instrument* or equipment or gadget* or app or apps or system or systems or solution* or software or service* or bundle or package* or aid* or assistan* or support* or self manag*)).ti,ab,kf. | 250,485 |
| 16 | ((schedul* or organi#ation* or productiv* or plann*) adj3 (technolog* or device* or product* or tool* or instrument* or equipment or gadget* or app or apps or system or systems or solution* or software or service* or bundle or package* or aid* or assistan* or support* or self manag*)).ti,ab,kf. | 188,959 |
| 17 | ((technolog* or digital or virtual or electronic) adj3 (intervention* or therap* or interactive)).ti,ab,kf. | 17,739 |
| 18 | (ehealth or e health or mhealth or m health or telehealth or tele health or telecare or tele care or telemed* or tele med* or telerehab* or tele rehab or in home).ti,ab,kf,jw. | 76,516 |
| 19 | (computer* or tablet* or handheld or hand held or touchscreen* or touch screen*).ti,ab,kf. | 427,695 |
| 20 | ((artificial* or machine or comput*) adj3 (intelligen* or learning or reasoning)).ti,ab,kf. | 122,805 |
| 21 | (telephone* or tele phone* or cellphone* or cell phone* or cellular phone* or cellular device* or smartphone* or smart phone* or smartwatch* or smart watch* or smarthome* or smart home*).ti,ab,kf. | 101,164 |
| 22 | (clock* or watch or watches or calendar* or agenda* or journal or journal?ing or diary or diaries or agenda*).ti,ab,kf. | 207,090 |
| 23 | (augmentative communicat* or alternative communicat* or text to speech or read aloud or voice command* or vocal command*).ti,ab,kf. | 2,591 |
| 24 | (((memor* or cognit* or mental or brain or intellect* or executive function) adj3 (train* or exercise* or challenge*)) and (game* or gaming or app or apps or application* or software* or website* or internet* or device* or tool*)).ti,ab,kf. | 4,160 |
| 25 | (prosthe* or orthot* or continence or incontinence or bed pan* or switch or switches or remote* or vehicle* or car or cars or van or vans or walker* or wheelchair* or wheeled or cane* or crutch* or scooter* or handrail* or grab bar*).ti,ab,kf,hw,jw. | 992,577 |
| 26 | ((eating or feeding or food* or drink* or wash* or bath* or shower* or toilet* or personal care or recreation* or leisur* or garden* or sport* or driving or walking or kitchen* or dishwash* or household or cleaning or seating or sleeping or body support or standing or dressing or undressing) adj5 (technolog* or device* or product* or tool* or instrument* or equipment or gadget* or app or apps or system or systems or solution* or software or service* or bundle or package* or aid* or assistan* or support* or self manag*)).ti,ab,kf,jw. | 162,015 |
| 27 | or/7-26 [Assistive Technology] | 3,438,366 |
| 28 | "Systematic Review"/ or Meta-Analysis/ or Network Meta-Analysis/ | 317,390 |
| 29 | "Review Literature as Topic"/ or Systematic Reviews as Topic/ or Meta-Analysis as Topic/ | 36,868 |
| 30 | exp Technology Assessment, Biomedical/ | 12,140 |
| 31 | ((systematic or scoping or rapid or qualitative or quantitative or integrative or umbrella or knowledge or evidence) adj3 (review or overview or synthesis)).ti,ab,kf,pt. | 381,101 |
| 32 | (technology assessment* or meta analys* or metaanalys*).ti,ab,kf,pt. | 305,959 |
| 33 | (prospero or medline or pubmed or embase or cinahl).ab. | 330,667 |
| 34 | (database* adj3 search*).ab. | 96,711 |
| 35 | or/28-34 [Knowledge Syntheses] | 674,861 |
| 36 | 6 and 27 and 35 | 3,416 |
